# A novel replication-deficient feline herpesvirus type 1 vector-based vaccine provides strong immune protection in cats

**DOI:** 10.1128/jvi.02188-25

**Published:** 2026-04-27

**Authors:** Wuchang Heng, Ziyan Zhou, Weiwei Lin, Xianglin Zhang, Ruibin Qi, Chenxi Wei, Qian Jiang, Hongtao Kang, Honglin Jia, Jiasen Liu

**Affiliations:** 1 State Key Laboratory of Animal Disease Control and Prevention, Harbin Veterinary Research Institute, Chinese Academy of Agricultural Sciences, Harbin, China; 2 Heilongjiang Research Center for Veterinary Biopharmaceutical Technology, Harbin Veterinary Research Institute, Chinese Academy of Agricultural Sciences, Harbin, China; University of Virginia, Charlottesville, Virginia, USA

**Keywords:** herpesvirus, vaccine safety, neutralizing antibody, FHV-1, FPV, FCV

## Abstract

Feline herpesvirus type 1 (FHV-1) is a member of the Alphaherpesvirinae subfamily, which includes important pathogens affecting humans and animals, such as herpes simplex virus types 1 and 2, pseudorabies virus, and Marek’s disease virus. While herpesviruses show promise as vaccine vectors for delivering foreign antigens, ensuring their safety is a significant concern that must be addressed alongside their efficacy. In this study, we report the development of a replication-defective FHV-1 ΔgL-based vaccine candidate, which is propagated exclusively in a specially modified F81 cell line. Vaccination of cats with the FHV-1 ΔgL-based candidate demonstrated a favorable safety profile and elicited protective immunity against FHV-1 challenge. More importantly, the FHV-1 ΔgL platform exhibits excellent efficacy in delivering the VP2 antigen of the feline panleukopenia virus (FPV), inducing high-titer neutralizing antibodies in cats. Furthermore, we evaluated a trivalent vaccine candidate, rQD1 ΔgL/gI/gE-VP2-VP1, in which an expression cassette for the feline calicivirus (FCV) VP1 was inserted to replace the gI/gE genes. Vaccination of cats with rQD1 ΔgL/gI/gE-VP2-VP1 produced a strong protective effect against challenges from FHV-1, FPV, and FCV. To support clinical manufacturing, we generated a stable cell line through site-specific integration of the gL gene into the Rosa26 safe-harbor locus of F81 cells. In conclusion, the FHV-1 ΔgL platform demonstrates safety, efficacy, and promising potential for future applications and serves as an example for constructing attenuated vaccines in alphaherpesviruses.

**IMPORTANCE:**

Herpesviruses are highly effective at presenting foreign antigens and serve as excellent viral vectors, yet their safety warrants careful attention. We developed a replication-defective feline herpesvirus type 1 (FHV-1) ΔgL vector platform. Using this platform, a bivalent vaccine (rQD1 ΔgL-VP2) and a trivalent vaccine (rQD1 ΔgL/gI/gE-VP2-VP1) were constructed. Both vaccines demonstrated robust immunoprotection and favorable safety profiles in immunized cats. Furthermore, to facilitate the application of this platform, a stable cell line with site-specific integration, F81-gL-Rosa26, was generated by inserting the gL gene into the Rosa26 safe-harbor locus of F81 cells. The strategy of the FHV-1 ΔgL platform also provides a reference for other members of the herpesvirus family.

## INTRODUCTION

Herpesviridae is a family of large, enveloped viruses with double-stranded DNA genomes ranging from 100 to 200 kb. The family comprises three subfamilies: Alphaherpesvirinae, Betaherpesvirinae, and Gammaherpesvirinae (1, 2). These viruses exhibit a broad host range, infecting humans, pigs, poultry, cats, and other species, often establishing lifelong infections and posing significant threats to human and animal health.

Herpesviruses have an envelope and infect host cells by fusing their envelope membrane with that of the host cells. This fusion can take place either within an endocytic vesicle or directly at the plasma membrane. The entry process is driven by a group of essential glycoproteins, which include glycoprotein D (gD), glycoprotein H (gH), glycoprotein L (gL), and glycoprotein B (gB) (1, 3). While gB, gH, and gL are conserved across herpesviruses, gD is specific to alphaherpesviruses and acts as a receptor-binding protein, facilitating the attachment of the virus to the host cell. The heterodimer formed by gL and gH serves as a core component of the herpesvirus membrane fusion machinery, working in concert with gB to mediate fusion between the viral envelope and the host cell membrane (4). Mutations in gL can impair its binding to gH, thereby reducing viral infectivity (5). Studies have demonstrated that the gH/gL complex not only plays a critical role in viral entry but also is essential for cell-to-cell spread of the virus (6). gL contains a chemokine-like structural motif, which mediates its interaction with the chemokine receptor of the host cells and may facilitate the spread of the virus (7, 8). However, the function of gL in cell entry may exhibit diversity across different herpesviruses. For instance, in herpes simplex virus (HSV-1 and HSV-2) and human herpesvirus 6, the complex formed by gL with gH is indispensable for viral entry into host cells (9, 10). In contrast, in bovine herpesvirus 4 (a gammaherpesvirus), gL promotes endocytosis during viral entry but is not essential for viral infectivity (11).

Currently, the development of viral vector-based vaccines has garnered significant attention (12–18). Herpesviruses are considered excellent candidates for use as vaccine vectors due to their large genome size, which enables the carriage of long gene fragments, and their ability to stimulate pro-inflammatory cytokines and induce type I interferons (19). Multiple attenuated herpesviral vaccine vectors have been developed by deleting virulence-associated genes and inserting heterologous genes (15, 17, 20–22). Replication-deficient viral vaccines have demonstrated efficacy in inducing neutralizing antibodies (NAbs) and cellular immune responses, thus providing immune protection (23–25). Replication-incompetent herpesvirus vectors have also been developed and previously evaluated for their ability to induce durable immune responses and protective efficacy (26–30).

Feline herpesvirus type 1 (FHV-1) infection causes severe conjunctivitis, corneal ulcers, sneezing, and ocular purulent discharge in cats, potentially resulting in blindness (31). Although there is scant evidence that vaccination during pregnancy causes harm, the currently available modified-live vaccines are not recommended for use in pregnant or immunocompromised cats per the 2024 World Small Animal Veterinary Association vaccination guidelines (32). In this study, we investigated the replication phenotype of the FHV-1 ΔgL mutant and assessed its feasibility for vaccine development. Additionally, we evaluated the safety and efficacy of this novel replication-defective virus as a vector platform by constructing two recombinant viruses: rQD1 ΔgL-VP2 and rQD1 ΔgL/gI/gE-VP2-VP1. The results of our study indicated that rQD1 ΔgL shows promising potential as a vaccine candidate against FHV-1 infection and as a vaccine vector for use in cats.

## RESULTS

### *In vitro* characterization of rQD1 ΔgL-VP2

**Fig 1:**
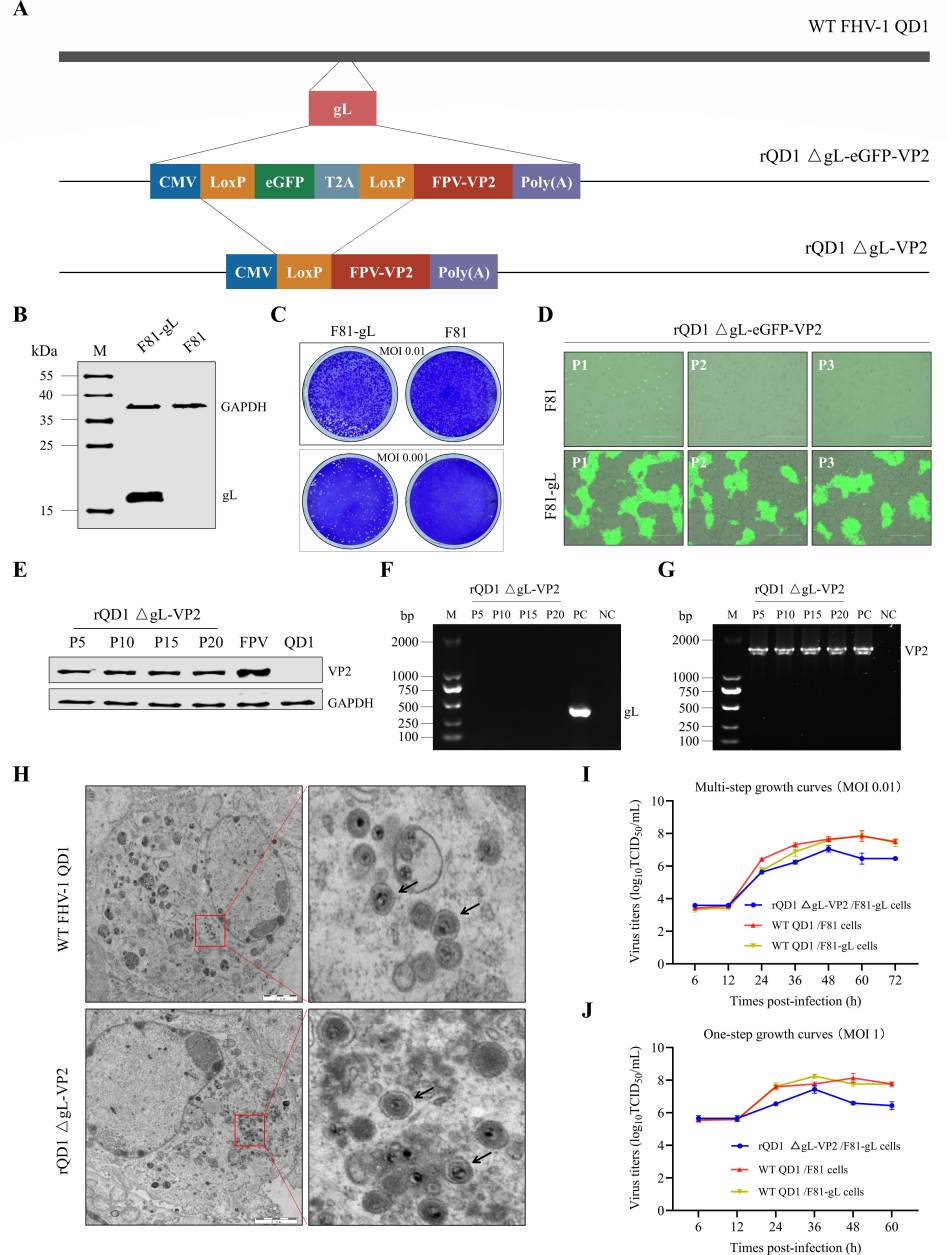
*In vitro* characterization of rQD1 ΔgL-VP2. (**A**) Construction of recombinant FHV-1 rQD1 ΔgL-VP2 expressing the FPV VP2 gene in the gL protein-stably expressing F81 cell line, F81-gL (**B**). (**C**) Plaque assay of WT FHV-1 QD1 infecting conventional F81 cells and F81-gL cells at MOIs of 0.01 and 0.001, respectively. (**D**) rQD1 ΔgL-eGFP-VP2 produced green fluorescent cytopathic effects (CPE) in F81 and F81-gL cells during serial passaging. Scale bar: 400 μm. (**E**) rQD1 ΔgL-VP2 was serially passaged in F81-gL cells up to P20. Western blot analysis confirmed stable VP2 expression at passages 5, 10, 15, and 20. Positive control: WT FPV-infected cells. Negative control: WT FHV-1 QD1-infected cells. (**F**) PCR detection of the gL gene in rQD1 ΔgL-VP2 at passages P5, P10, P15, and P20. Positive control: WT FHV-1 genome DNA. Negative control: WT FPV genome DNA. (**G**) PCR detection of the VP2 gene in rQD1 ΔgL-VP2 at passages P5, P10, P15, and P20. Positive control: pcDNA3.1 eGFP-FPV VP2. Negative control: WT FHV-1 genome DNA. (**H**) Morphology of rQD1 ΔgL-VP2 virions observed by TEM at 48 h after infecting conventional F81 cells at an MOI of 1. Scale bar: 2 µm. Black arrows indicate mature viral particles. (**I**) Multi-step growth curves of WT FHV-1 QD1 and rQD1 ΔgL-VP2. F81 or F81-gL cells were infected at an MOI of 0.01. (**J**) One-step growth curves of WT FHV-1 QD1 and rQD1 ΔgL-VP2. F81 or F81-gL cells were infected at an MOI of 1. The data are expressed as mean ± SD.

A recombinant virus, rQD1 ΔgL-VP2, was successfully constructed using the F81-gL cell line (which stably expresses gL) and the CRISPR/Cas9 system combined with the Cre-LoxP method (Fig. 1A and B). In this virus, the gL gene of the FHV-1 QD1 strain was replaced with an expression cassette for the VP2 gene from the feline panleukopenia virus (FPV) SR strain. To examine the role of gL in viral replication, we performed plaque assays by infecting both wild-type F81 cells and F81-gL cells with the rQD1 ΔgL-VP2 strain at multiplicities of infection (MOIs) of 0.01 and 0.001. Compared to those on F81-gL cells, plaques formed on the wild-type cells were much smaller, suggesting that gL is essential for efficient viral cell-to-cell spread (Fig. 1C). The recombinant virus rQD1 ΔgL-eGFP-VP2 was used to infect wild-type F81 cells at a MOI of 0.01 and was passaged to the third generation (P3). The results indicated that rQD1 ΔgL-eGFP-VP2 exhibited only single-cycle replication in wild-type F81 cells (Fig. 1D). To assess the stability of the recombinant rQD1 ΔgL-VP2 strain, the virus was serially passaged in F81-gL cells up to P20 at an MOI of 0.01. Western blot analysis confirmed stable expression of the VP2 protein (Fig. 1E), and PCR detection verified the successful knockout of the gL gene (Fig. 1F). This indicates that rQD1 ΔgL-VP2 is a stable virus strain, free of wild-type virus, and that the VP2 gene was maintained throughout passaging (Fig. 1G). Transmission electron microscopy (TEM) analysis of the rQD1 ΔgL-VP2 recombinant virus produced in wild-type F81 cells revealed classical FHV-1 virion morphology (Fig. 1H), suggesting that the gL protein does not affect virion assembly. Subsequently, we evaluated the one-step (MOI 1) and multi-step (MOI 0.01) growth curves of QD1 and rQD1 ΔgL-VP2 (Fig. 1I and J). The replication capacity of the wild-type QD1 strain was similar in wild-type F81 cells and F81-gL cells. rQD1 ΔgL-VP2 exhibited slightly attenuated replication, with a peak virus titer reaching 10^7.5^ TCID_50_/mL. Collectively, these data demonstrate that the gL gene is essential for the replication of FHV-1. The deletion of the gL gene results in a single-cycle replication phenotype in wild-type F81 cells but does not affect virion assembly. Furthermore, rQD1 ΔgL-VP2 exhibits favorable genetic stability.

### rQD1 ΔgL-VP2 confers effective immunoprotection against FHV-1 and elicits high levels of neutralizing antibodies against FPV in cats

**Fig 2:**
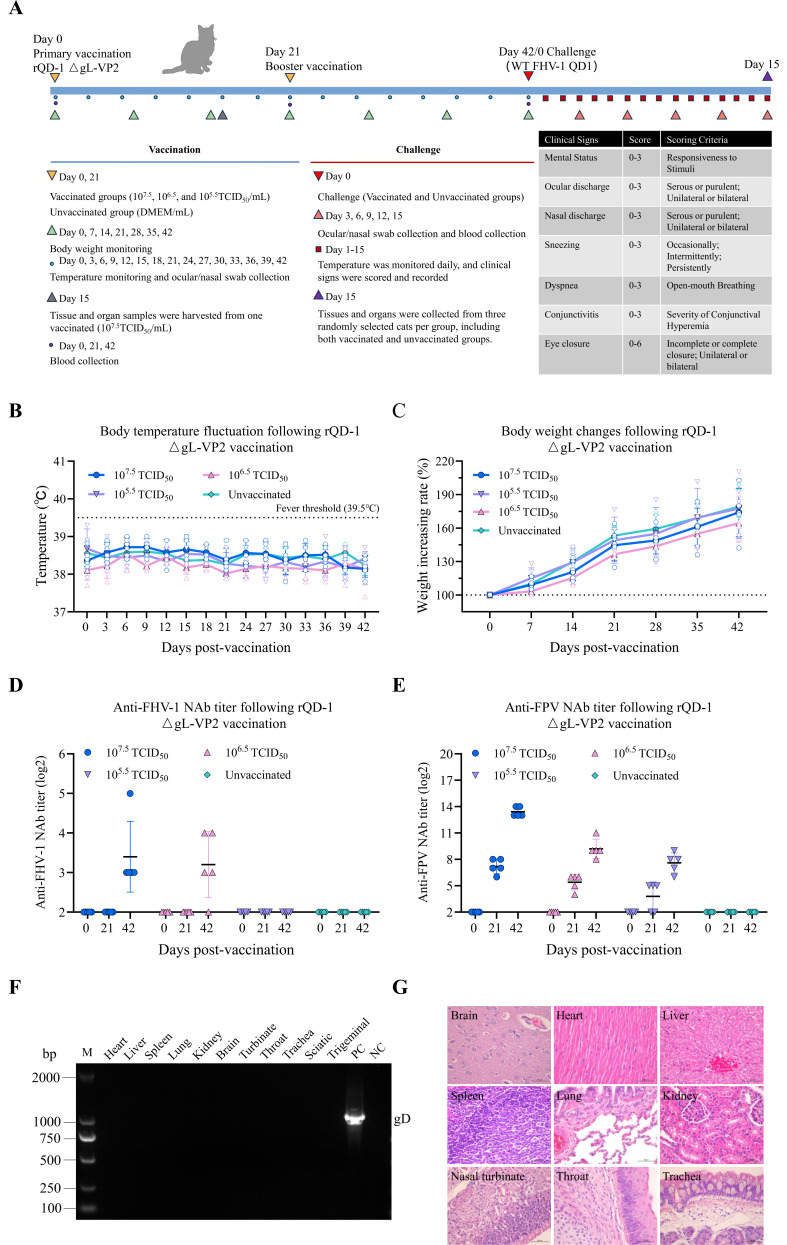
rQD1 ΔgL-VP2 induces NAbs against FHV-1 and FPV and is safe *in vivo*. (**A**) Cats received two intramuscular doses of rQD1 ΔgL-VP2 (10^7.5^, 10^6.5^, and 10^5.5^ TCID_50_) 42 days apart, followed by WT FHV-1 QD1 challenge 15 days post-immunization. Body temperature (**B**) was monitored every 3 days, and body weight (**C**) was measured every 7 days during the immunization period. NAb titers against FHV-1 (**D**) and FPV (**E**) were assessed on days 0, 21, and 42. Tissue samples from a vaccinated cat were analyzed for the gD gene by PCR (**F**), and major organs were examined by histopathology (**G**). Scale bar: 50 μm. The data are expressed as mean ± SD.

Cats were inoculated with rQD1 ΔgL-VP2 at different doses (10^7.5^, 10^6.5^, and 10^5.5^ TCID_50_) over an immunization period of 42 days, followed by a challenge period with FHV-1 lasting 15 days (Fig. 2A). Throughout the 42 day post-inoculation period, both vaccinated and unvaccinated groups exhibited normal fluctuations in body temperature (Fig. 2B) and showed an overall increase in body weight (Fig. 2C). All cats remained healthy and displayed no clinical signs of illness. No viral shedding was detected in ocular and nasal swabs using qPCR (data not shown). NAb assays (Fig. 2D and E) revealed that the group vaccinated with 10^7.5^ TCID_50_ of vaccine developed anti-FHV-1 NAb titers of at least 3 log2 and very high anti-FPV NAb titers of at least 13 log2 by the end of the immunization period. In the 10^6.5^ TCID_50_ group, four cats produced FHV-1 NAb titers of at least 3 log2, along with relatively high anti-FPV NAb titers of at least 8 log2. Conversely, all cats in the 10^5.5^ TCID_50_ group exhibited FHV-1 NAb titers of less than 3 log2 but still showed relatively high levels of anti-FPV NAbs of at least 6 log2. These findings highlight the effectiveness of rQD1 ΔgL-VP2 in delivering the VP2 antigen. PCR analysis of tissue samples from one randomly selected cat in the 10^7.5^ TCID_50_ group found no evidence of viral invasion (Fig. 2F). Histopathological examination of tissues from the vaccinated group, including the heart, liver, spleen, lung, kidney, brain, nasal turbinate, throat, and trachea, revealed no abnormalities (Fig. 2G). Overall, these data demonstrate that rQD1 ΔgL-VP2 has a favorable safety profile and induces high levels of NAbs against FPV.

**Fig 3:**
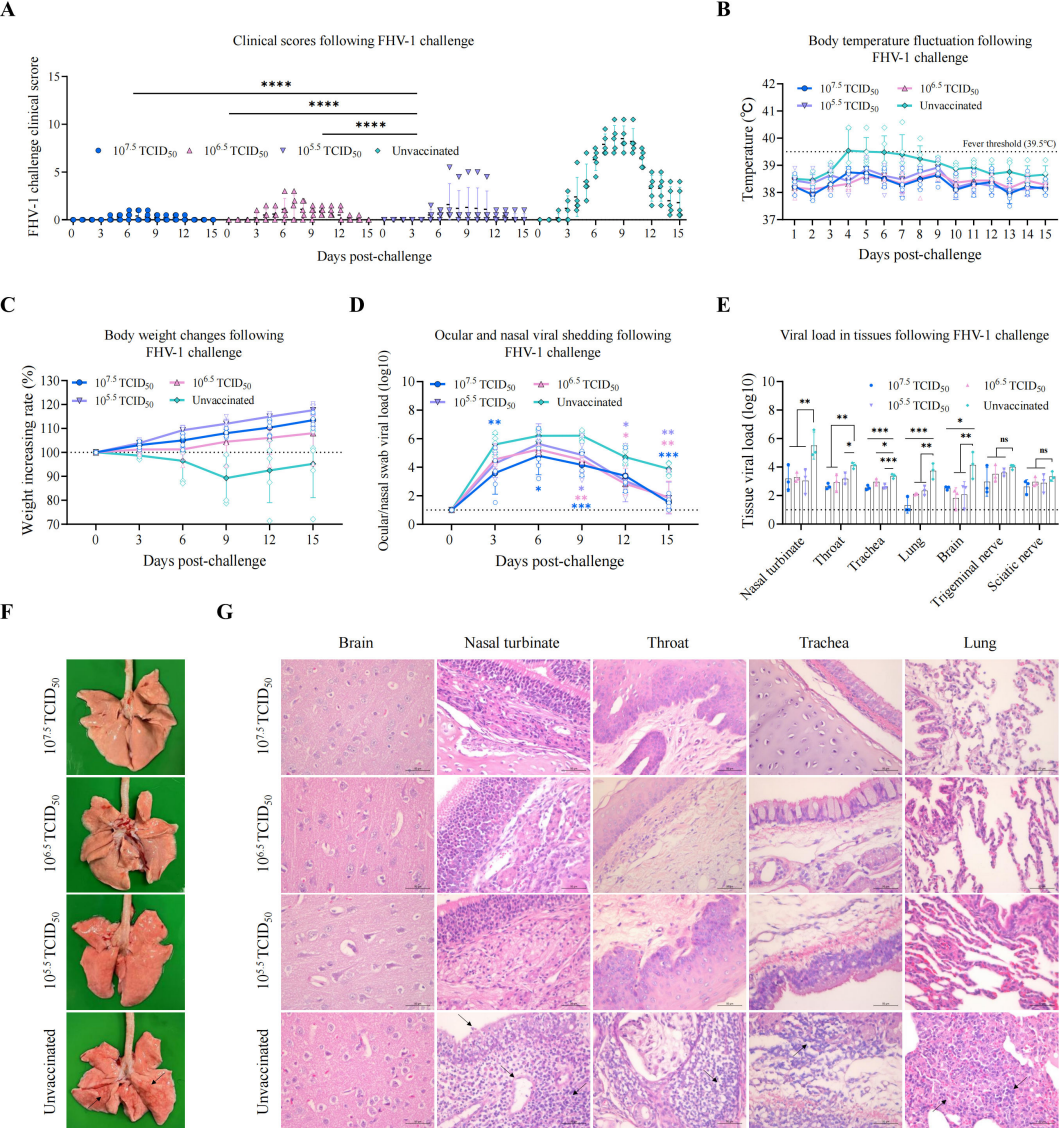
rQD1 ΔgL-VP2 confers protective immunity against FHV-1 in cats. Cats immunized with rQD1 ΔgL-VP2 were challenged with 10^5.5^ TCID_50_/0.5 mL of WT FHV-1 QD1 for 15 days. (**A**) Clinical signs were scored and recorded daily. (**B**) Body temperature was monitored daily, while body weight (**C**) was measured, and combined ocular/nasal swab samples (**D**) were collected every 3 days for qPCR analysis of viral load (copies/20 ng). (**E**) On day 15, all cats were euthanized. Tissue samples were harvested for qPCR detection of viral load (copies/100 ng). (**F**) Lungs were examined for gross lesions during necropsy. Black arrows indicate areas of pathological changes. (**G**) Histopathological analysis of the brain, nasal turbinate, throat, trachea, and lung. Scale bar: 50 μm. Black arrows indicate areas of pathological changes. (**D, E**) The dashed line denotes the limit of detection. Statistical analysis was performed using one-way ANOVA followed by Tukey’s multiple-comparison test. The significance level was defined as *P < 0.05, **P < 0.01, ***P < 0.001, and ****P < 0.0001. ns, non-significant. The data are expressed as mean ± SD.

Following the FHV-1 challenge, clinical sign scores were recorded daily for both the vaccinated and unvaccinated groups (Fig. 3A). The vaccinated group exhibited only mild serous ocular or nasal discharge, whereas the unvaccinated group developed severe signs including purulent ocular and nasal discharge, persistent sneezing, dyspnea, severe conjunctivitis, and even bilateral eye closure, demonstrating a marked contrast to the vaccinated group. Regarding body temperature, the unvaccinated group developed fever (exceeding 39.5°C) post-challenge, with the mean temperature peaking on day 4 before gradually returning to normal, while the vaccinated group maintained temperatures within the normal range throughout (Fig. 3B). In terms of body weight, the unvaccinated group experienced weight loss after challenge until day 9, when gradual recovery began, whereas the vaccinated group showed continuous weight gain (Fig. 3C). Viral shedding assessed via ocular and nasal swabs revealed significantly lower levels in the vaccinated groups. Specifically, the 10^7.5^ TCID_50_ group showed significantly reduced shedding on days 3, 6, 9, and 12 compared to the unvaccinated group, while the 10^6.5^ and 10^5.5^ TCID_50_ groups exhibited significant reduction on days 9, 12, and 15 (Fig. 3D). Viral loads in the brain, nasal turbinate, throat, trachea, and lungs of vaccinated cats were significantly lower than those in the unvaccinated group. Although mean viral loads in the sciatic and trigeminal nerves were lower in vaccinated cats, the differences were not statistically significant (Fig. 3E). Macroscopic examination of lungs post-necropsy revealed diffuse hemorrhage on the surface of lungs from the unvaccinated group, whereas no significant pathological changes were observed in the vaccinated group (Fig. 3F). Histopathological analysis revealed severe lesions in tissues other than the brain in the unvaccinated group, characterized by degeneration and necrosis of the mucosal epithelial cells in the nasal turbinates, extensive inflammatory cell infiltration in the mucosal and submucosal layers, edema in the lamina propria, prominent inflammatory cell infiltration in the submucosal layer of the throat, inflammatory cell infiltration in the mucosal layer of the trachea, and widening of the alveolar septa with visible inflammatory cell infiltration in the lungs. In contrast, tissues from the vaccinated group exhibited normal histology (Fig. 3G). Collectively, these data demonstrate that rQD1 ΔgL-VP2 elicits effective immune protection against FHV-1 challenge.

### *In vitro* characterization of rQD1 ΔgL/gI/gE-VP2-VP1

**Fig 4:**
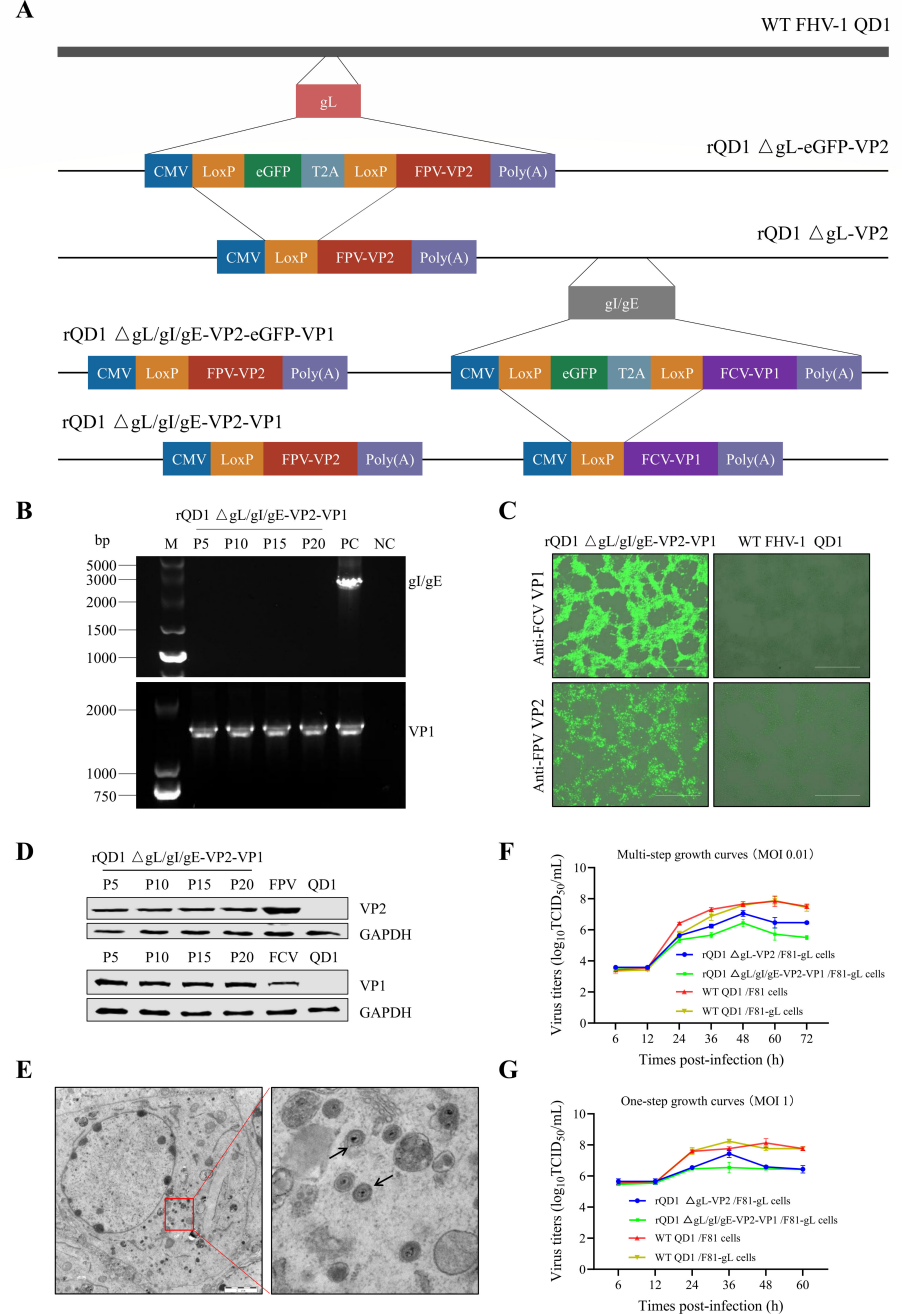
*In vitro* characterization of rQD1 ΔgL/gI/gE-VP2-VP1. (**A**) Construction strategy of the recombinant FHV-1 rQD1 ΔgL/gI/gE-VP2-VP1 in F81-gL cells. (**B**) PCR detection of the deleted gI/gE gene region and the inserted VP1 gene in rQD1 ΔgL/gI/gE-VP2-VP1 at passages P5, P10, P15, and P20. Positive controls: WT FHV-1 genomic DNA (for gI/gE) or pVAX1-VP1 (for VP1). Negative control: sterile water. (**C**) IFA confirmed VP2 and VP1 expression in infected F81-gL cells with rQD1 ΔgL/gI/gE-VP2-VP1. Scale bar: 400 μm. (**D**) The stability of VP2 and VP1 expression in infected cells at passages P5, P10, P15, and P20 was analyzed by Western blot. Positive controls: WT FPV-infected cells (for VP2) or WT FCV-infected cells (for VP1). Negative control: WT FHV-1 QD1-infected cells. (**E**) Virion morphology of rQD1 ΔgL/gI/gE-VP2-VP1 observed by TEM at 48 h post-infection after infecting conventional F81 cells at an MOI of 1. Scale bar: 2 μm. Black arrows indicate mature viral particles. (**F**) Multi-step growth curve of rQD1 ΔgL/gI/gE-VP2-VP1. F81-gL cells were infected at an MOI of 0.01. (**G**) One-step growth curve of rQD1 ΔgL/gI/gE-VP2-VP1. F81-gL cells were infected at an MOI of 1. The data are expressed as mean ± SD.

On the basis of rQD1 ΔgL-VP2, the gI/gE genes were targeted for knockout and replaced with a feline calicivirus (FCV) VP1 (without the LC region) expression cassette, resulting in the construction of rQD1 ΔgL/gI/gE-VP2-VP1 (Fig. 4A). After serial passaging up to P20, PCR analysis confirmed the deletion of the gI/gE genes and the presence of the VP1 gene in the recombinant virus (Fig. 4B). Furthermore, sequencing results of the exogenous VP2 and VP1 genes revealed no mutations during the serial passaging of the recombinant virus. Immunofluorescence assay (IFA) and Western blot analyses further demonstrated the expression of both foreign genes, VP2 and VP1, in F81-gL cells infected with the recombinant virus (Fig. 4C and D). Additionally, transmission electron microscopy of the recombinant virus revealed typical FHV-1 virion morphology (Fig. 4E). Furthermore, growth curve analysis demonstrated that the replication capacity of rQD1 ΔgL/gI/gE-VP2-VP1 was lower than that of rQD1 ΔgL-VP2 and the QD1 strain, with a peak titer of 10^6.67^ TCID_50_/mL (Fig. 4F and G). These data collectively demonstrate the stability of rQD1 ΔgL/gI/gE-VP2-VP1.

### rQD1 ΔgL/gI/gE-VP2-VP1 confers effective immunoprotection against FHV-1, FPV, and FCV in cats

**Fig 5:**
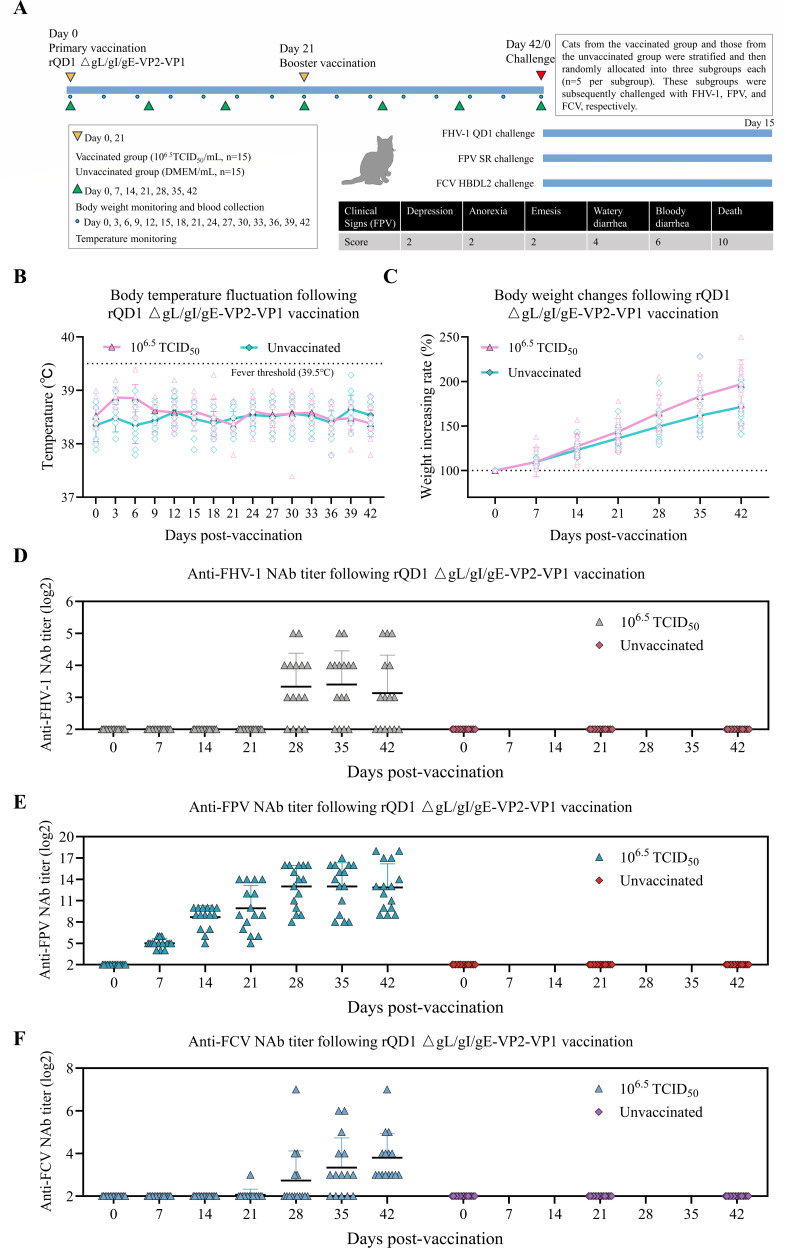
rQD1 ΔgL/gI/gE-VP2-VP1 elicits NAbs against FHV-1, FPV, and FCV. (**A**) Cats received two doses of rQD1 ΔgL/gI/gE-VP2-VP1 (10^6.5^ TCID_50_) 42 days apart. At 15 days post-immunization, they were challenged with WT FHV-1 QD1, FPV SR, or FCV HBDL2. (**B**) Body temperature was monitored every 3 days during the immunization period. (**C**) Body weight was measured every 7 days. NAb titers against FHV-1 (**D**), FPV (**E**), and FCV (**F**) in vaccinated and unvaccinated groups. The data are expressed as mean ± SD.

Cats were immunized with rQD1 ΔgL/gI/gE-VP2-VP1 at a dose of 10^6.5^ TCID_50_ (*n* = 15), while the control group received an equivalent volume of DMEM (*n* = 15). Following the immunization period, vaccinated and unvaccinated cats were each randomly divided into three subgroups (*n* = 5 per group) for challenge studies with FHV-1, FPV, and FCV (Fig. 5A). Throughout the immunization period, body temperature fluctuated within the normal range and body weight increased steadily (Fig. 5B and C). All cats remained clinically healthy without any adverse signs. The vaccinated group developed NAbs against FHV-1, FPV, and FCV (Fig. 5D through F). NAbs against FHV-1 and FCV were detectable only after the booster immunization. Notably, NAbs against FPV were detected as early as 1 week after the primary immunization. The NAb titers gradually increased and enhanced sharply 1 week after the booster immunization, eventually stabilizing at a maximum titer of up to 18 log2.

**Fig 6:**
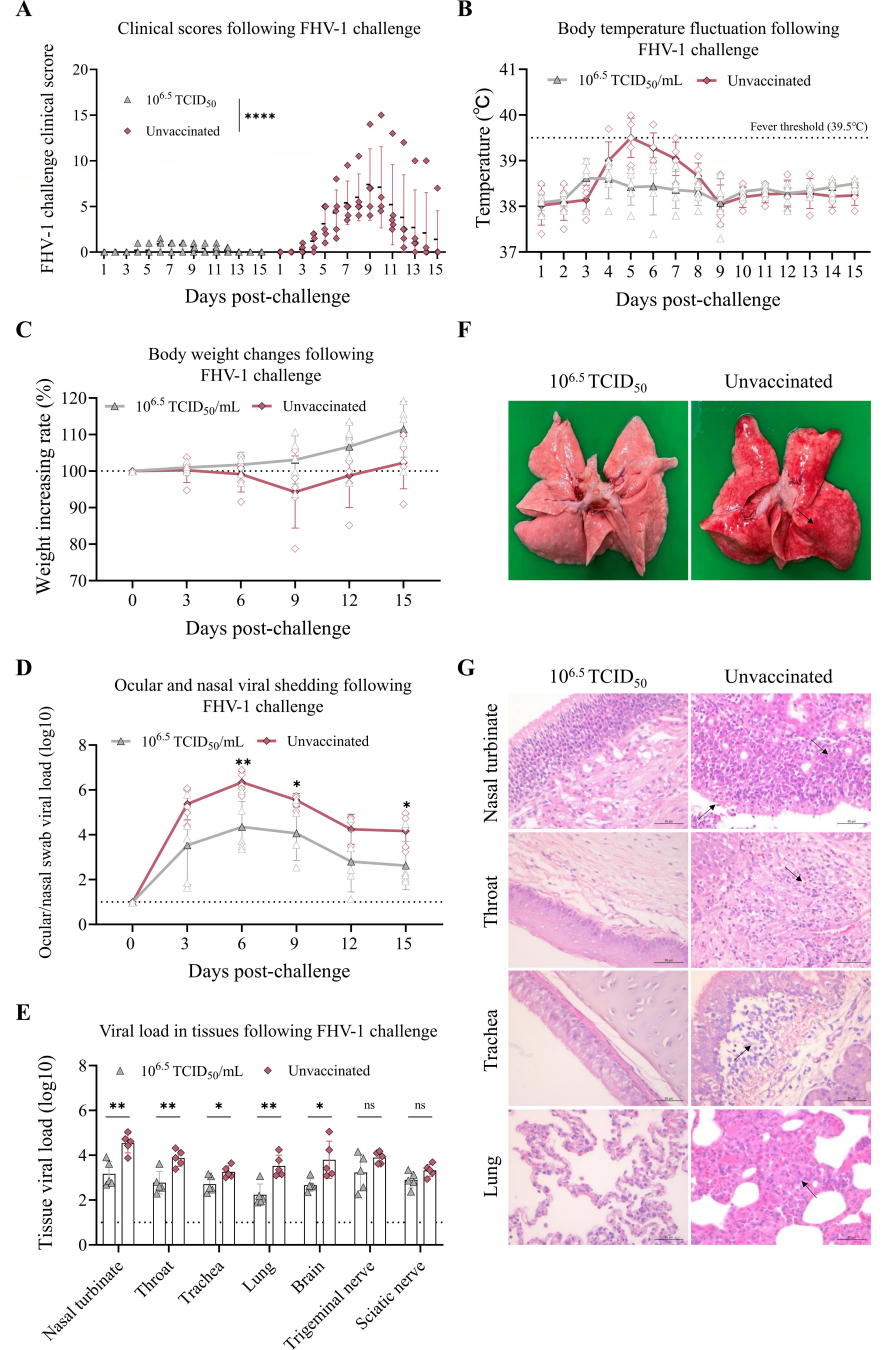
rQD1 ΔgL/gI/gE-VP2-VP1 confers protective immunity against FHV-1 in cats. Cats immunized with rQD1 ΔgL/gI/gE-VP2-VP1 were challenged with 10^5.5^ TCID_50_/0.5 mL of WT FHV-1 QD1 for 15 days. (**A**) Clinical signs were scored and recorded daily. (**B**) Body temperature was monitored daily, while body weight (**C**) was measured, and combined ocular/nasal swab samples (**D**) were collected every 3 days for qPCR analysis of viral load (copies/20 ng). (**E**) On day 15, all cats were euthanized. Tissue samples were harvested for qPCR detection of viral load (copies/100 ng). (**F**) Lungs were examined for gross lesions during necropsy. Black arrows indicate areas of pathological changes. (**G**) Histopathological analysis of the nasal turbinate, throat, trachea, and lung. Scale bar: 50 μm. Black arrows indicate areas of pathological changes. (**D, E**) The dashed line denotes the limit of detection. Statistical analysis was performed using an unpaired *t*-test. The significance level was defined as *P < 0.05 and **P < 0.01. ns, non-significant. The data are expressed as mean ± SD.

Following the FHV-1 challenge, the vaccinated group exhibited only mild clinical signs compared to the unvaccinated group (3/5 cats showed minimal serous ocular discharge). In contrast, all cats in the unvaccinated group developed a range of clinical signs, including abundant serous or purulent ocular and nasal discharges, persistent sneezing, dyspnea, conjunctivitis, and even eyelid closure (Fig. 6A). Transient elevation in body temperature (exceeding 39.5°C) was observed in the unvaccinated group on days 4, 5, and 6 post-challenge, whereas the vaccinated group maintained normal body temperature fluctuations (Fig. 6B). The unvaccinated group experienced weight loss post-challenge, with recovery beginning on day 9, while the vaccinated group showed steady weight gain unaffected by viral infection (Fig. 6C). Viral shedding in ocular and nasal secretions was significantly lower in the vaccinated group compared to the unvaccinated group on days 6, 9, and 15 post-challenge (Fig. 6D). Furthermore, viral loads in the brain, nasal turbinate, throat, trachea, and lungs were significantly reduced in the vaccinated group, whereas no statistically significant differences were observed in the sciatic and trigeminal nerves (Fig. 6E). Macroscopic examination of the lungs revealed hemorrhagic lesions in the unvaccinated group, whereas no gross pathological changes were observed in the vaccinated group (Fig. 6F). Histopathological analysis demonstrated that in the unvaccinated group, the nasal concha exhibited abundant neutrophils within the nasal secretions, accompanied by degeneration and necrosis of mucosal epithelial cells and inflammatory cell infiltration. The throat revealed inflammatory cell infiltration in the lamina propria, while the trachea displayed similar inflammatory cell infiltration in the lamina propria. In the lungs, there was moderate widening of local alveolar septa with inflammatory cell infiltration observed within these areas, as well as perivascular inflammatory cell infiltration. In contrast, the vaccinated group showed an absence of any pathological alterations (Fig. 6G).

**Fig 7:**
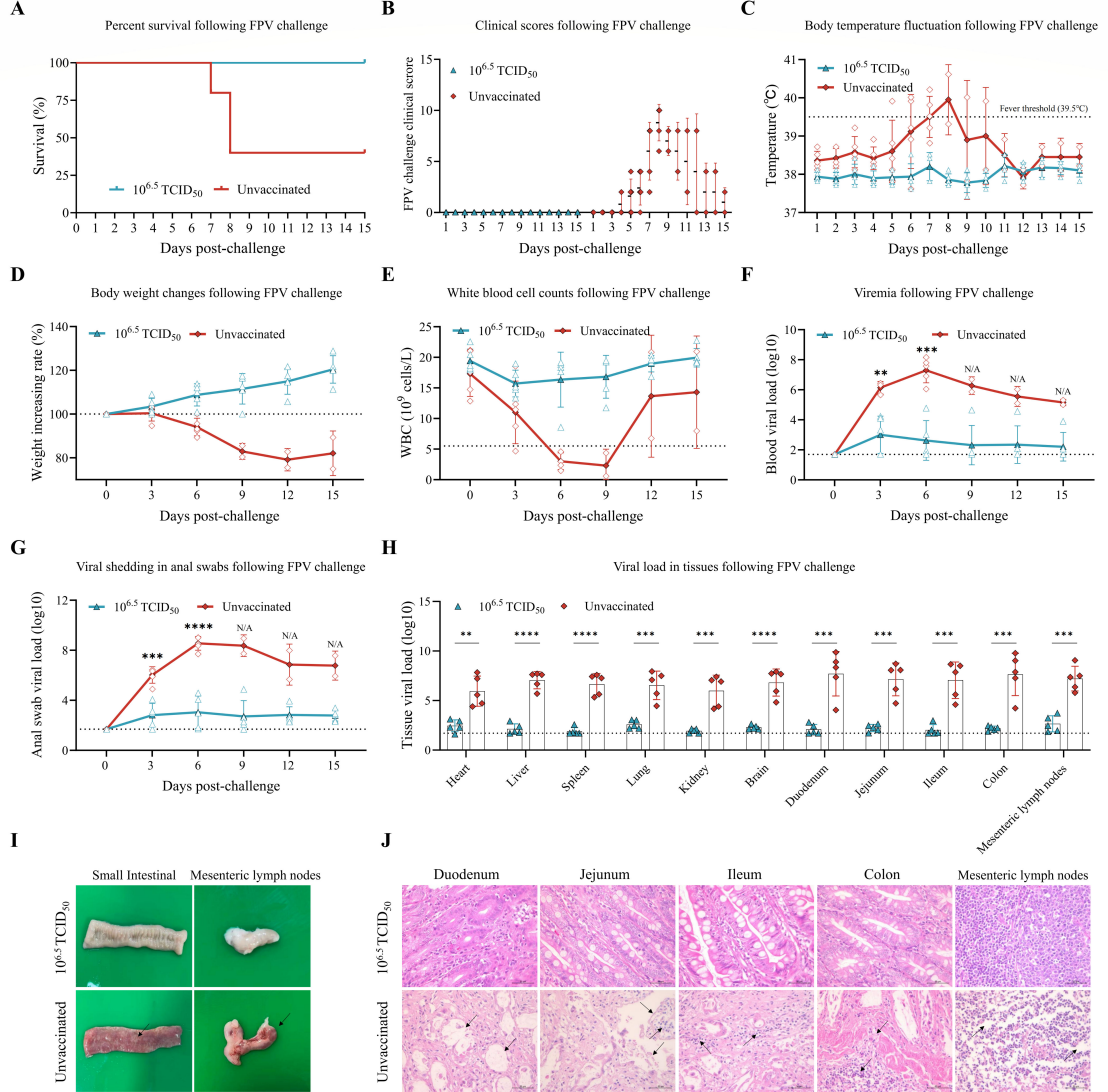
rQD1 ΔgL/gI/gE-VP2-VP1 confers protective immunity against FPV in cats. Cats immunized with rQD1 ΔgL/gI/gE-VP2-VP1 were challenged with 10^8^ TCID_50_/mL of WT FPV SR for 15 days. (**A**) Survival curves were plotted and monitored until day 15. (**B**) Clinical signs were scored and recorded daily throughout the challenge period. (**C**) Body temperature was monitored daily. (**D**) Body weight was measured every 3 days. (**E**) Anti-coagulated blood was collected every 3 days for white blood cell (WBC) count monitoring. (**F**) Viral load (copies/20 ng) in blood was detected by qPCR every 3 days. (**G**) Anal swabs were collected every 3 days for viral load (copies/20 ng) detection by qPCR. (**H**) Tissue samples were collected for viral load (copies/100 ng) detection by qPCR. (**I**) Gross lesions in the small intestine and mesenteric lymph nodes were observed during necropsy. Black arrows indicate areas of pathological changes. (**J**) Histopathological analysis of the duodenum, jejunum, ileum, colon, and mesenteric lymph nodes. Scale bar: 50 μm. Black arrows indicate areas of pathological changes. (**F–H**) The dashed line denotes the limit of detection. Statistical analysis was performed using an unpaired *t*-test. The significance level was defined as **P < 0.01, ***P < 0.001, and ****P < 0.0001. N/A, statistical analysis was not performed due to insufficient sample size. The data are expressed as mean ± SD.

Following the FPV challenge, three unvaccinated cats succumbed on days 7 and 8 post-challenge, resulting in a mortality rate of 60%. In contrast, all cats in the vaccinated group survived (100% survival) (Fig. 7A). Unvaccinated cats exhibited severe clinical signs, including depression, vomiting, diarrhea, hemorrhagic stool, and death. Conversely, no clinical signs were observed in the vaccinated group, with all cats maintaining a healthy state (Fig. 7B). Regarding body temperature, unvaccinated cats developed hyperthermia (exceeding 39.5°C) from day 5 to day 10. The two surviving unvaccinated cats showed a decrease in temperature, returning to the normal range after day 11. The vaccinated group maintained stable temperature fluctuations within the normal range (Fig. 7C). In terms of body weight, unvaccinated cats experienced weight loss until death, with the two survivors showing a slight recovery only after day 12. Vaccinated cats exhibited steady weight gain (Fig. 7D). Total white blood cell counts in unvaccinated cats decreased rapidly post-challenge until death; the surviving cats showed an increase to within the normal range (5.5–19.5 × 10^9^/L) after day 9 (Fig. 7E). Viral shedding loads in rectal swabs and viral loads in whole blood were significantly lower in the vaccinated group compared to the unvaccinated group on days 3 and 6, indicating a substantial reduction in viremia. Statistical analysis was not applicable for days 9, 12, and 15 due to the survival of only two cats in the unvaccinated group (Fig. 7F and G). Viral loads in the heart, liver, spleen, lungs, kidney, brain, duodenum, jejunum, ileum, colon, and mesenteric lymph nodes were significantly lower in the vaccinated group than in the unvaccinated group (Fig. 7H). Necropsy examination of the small intestine and mesenteric lymph nodes revealed thinned intestinal walls with extensive hemorrhage and widespread hemorrhage in the mesenteric lymph nodes in unvaccinated cats. No abnormal pathological changes were observed in the vaccinated group (Fig. 7I). Histopathological analysis revealed that in the unvaccinated group, there was intestinal villous atrophy, necrosis of mucosal epithelial cells, and inflammatory cell infiltration. The mesenteric lymph nodes exhibited extensive lymphoid depletion within the follicles. In contrast, the vaccinated group displayed only minimal inflammatory cell infiltration in the intestinal lamina propria (Fig. 7J).

**Fig 8:**
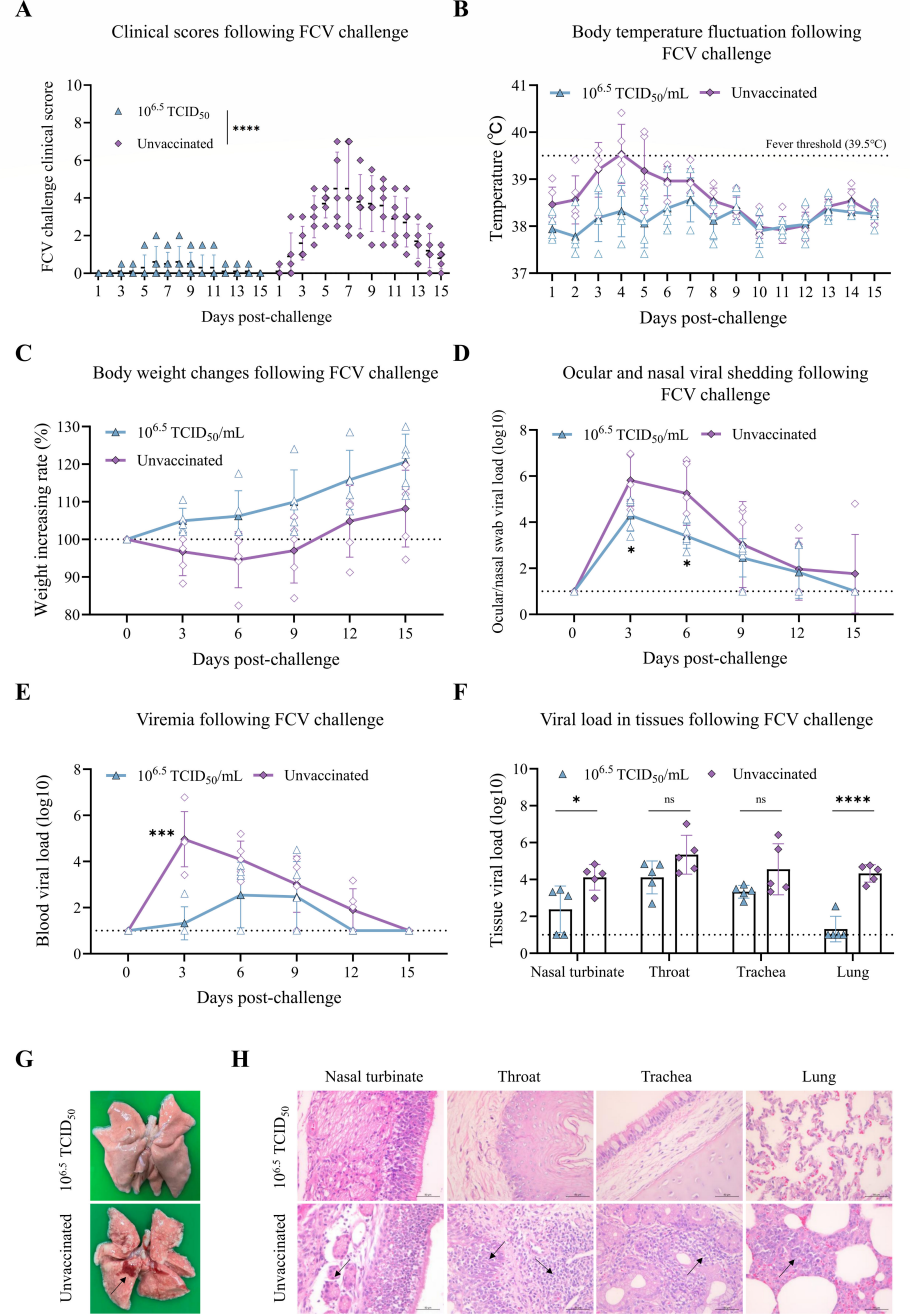
rQD1 ΔgL/gI/gE-VP2-VP1 confers protective immunity against FCV in cats. Cats immunized with rQD1 ΔgL/gI/gE-VP2-VP1 were challenged with 10^8^ TCID_50_/mL of WT FCV HBDL2 for 15 days. (**A**) Clinical signs were scored and recorded daily. (**B**) Body temperature was monitored daily, while body weight (**C**) was measured, and combined ocular/nasal swab samples (**D**) were collected every 3 days for qPCR analysis of viral load (copies/20 ng). (**E**) Viral load (copies/20 ng) in blood was detected by qPCR every 3 days. (**F**) On day 15, all cats were euthanized. Tissue samples were harvested for qPCR detection of viral load (copies/100 ng). (**G**) Lungs were examined for gross lesions during necropsy. Black arrows indicate areas of pathological changes. (**H**) Histopathological analysis of the nasal turbinate, throat, trachea, and lung. Scale bar: 50 μm. Black arrows indicate areas of pathological changes. (**D–F**) The dashed line denotes the limit of detection. Statistical analysis was performed using an unpaired *t*-test. The significance level was defined as *P < 0.05, ***P < 0.001, and ****P < 0.0001. ns, non-significant. The data are expressed as mean ± SD.

Following the FCV challenge, all cats in the unvaccinated group developed typical clinical signs (severe oral ulcers, ocular and nasal discharges, etc.), with clinical scores significantly higher than those in the vaccinated group (Fig. 8A). In contrast, cats in the vaccinated group maintained stable body temperatures and exhibited steady weight gain post-challenge. In the unvaccinated group, body temperatures increased (exceeding 39.5°C) between days 0 and 4, followed by gradual recovery, while weight loss occurred between days 0 and 6 before they started to regain weight (Fig. 8B and C). Viral shedding in ocular and nasal secretions was significantly lower in the vaccinated group compared to the unvaccinated group on days 3 and 6 (Fig. 8D). Additionally, the vaccinated group showed significantly lower viral loads in whole blood on day 3, indicating that vaccination mitigated viremia (Fig. 8E). Viral loads in tissues (nasal turbinate and lungs) were significantly reduced in the vaccinated group, demonstrating that vaccination greatly limited viral invasion into the lungs (Fig. 8F). Upon necropsy, no significant pulmonary lesions were observed in the vaccinated group, whereas the unvaccinated group exhibited varying degrees of pulmonary hemorrhage (Fig. 8G). Histopathological analysis revealed that in the unvaccinated group, inflammatory cell infiltration was observed in the lamina propria of the nasal conchal mucosa. The throat exhibited inflammatory cell infiltration within the mucosal layer, accompanied by degeneration and necrosis of the mucosal epithelial cells. The trachea showed inflammatory cell infiltration in the lamina propria, while the lungs demonstrated moderate widening of the alveolar walls with associated inflammatory cell infiltration. In contrast, no pathological alterations were observed in the vaccinated group (Fig. 8H). These data indicate that rQD1 ΔgL/gI/gE-VP2-VP1 confers effective immune protection in cats against FHV-1, FPV, and FCV.

### The F81-gL-Rosa26 cell line exhibits promising potential for clinical application

**Fig 9:**
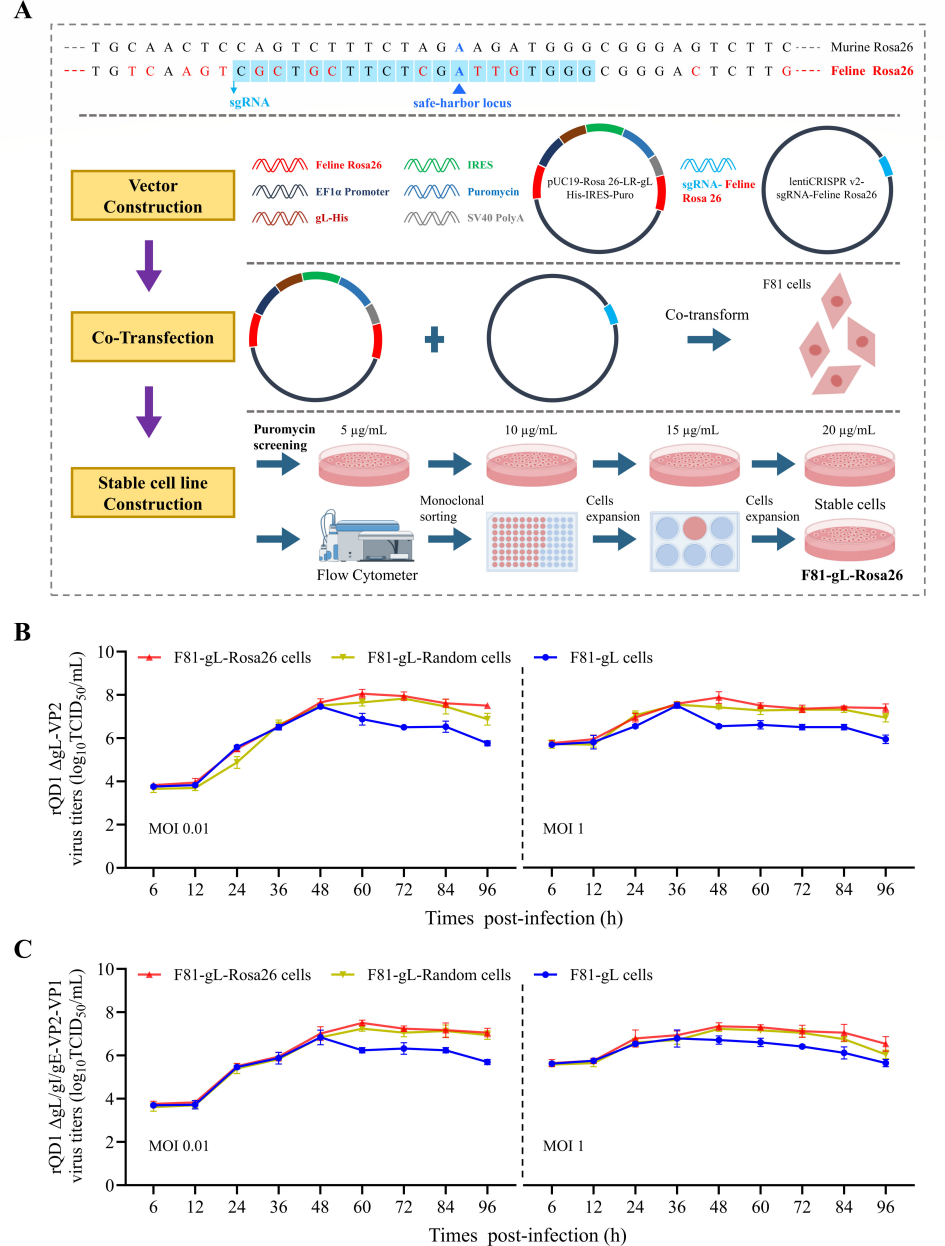
The F81-gL-Rosa26 cell line exhibits promising potential for clinical application. (**A**) Construction strategy of the F81-gL-Rosa26 cell line. One-step (MOI 1) and multi-step (MOI 0.01) growth curves of rQD1 ΔgL-VP2 (**B**) and rQD1 ΔgL/gI/gE-VP2-VP1 (**C**) in F81-gL, F81-gL-Rosa26, and F81-gL-Random cell lines. The data are expressed as mean ± SD.

While lentivirus-generated cell lines are often suitable for laboratory research, clinical production requires cell lines meeting higher safety standards. To this end, we first identified a safe genomic locus by homology alignment and sequencing of the feline Rosa26 gene homolog, thereby enabling the construction of the stable, site-specifically integrated cell line F81-gL-Rosa26 using the CRISPR/Cas9 system (Fig. 9A). Additionally, a linearized recombinant plasmid lacking feline Rosa26 homology arms was transfected into F81 cells. Following puromycin selection, the random integration cell line F81-gL-Random was successfully established. Subsequently, we evaluated the one-step (MOI 1) and multi-step (MOI 0.01) growth curves of the recombinant viruses rQD1 ΔgL-VP2 and rQD1 ΔgL/gI/gE-VP2-VP1 in the F81-gL, F81-gL-Rosa26, and F81-gL-Random cell lines (Fig. 9B and C). The results indicated that both the F81-gL-Rosa26 and F81-gL-Random cell lines efficiently supported robust virus production, yielding higher viral titers than the laboratory-stage F81-gL cell line. Importantly, the F81-gL-Rosa26 cell line, which features targeted integration into a defined safe-harbor locus, is better suited for safe clinical manufacturing. This advancement significantly enhances the applicability of the two candidate vaccine strains, rQD1 ΔgL-VP2 and rQD1 ΔgL/gI/gE-VP2-VP1, investigated in this study.

## DISCUSSION

Replication-defective viral vaccines combine the convenience and immunogenicity of live-attenuated platforms with the safety of inactivated or subunit vaccines (33). Therefore, this study aims to develop an effective replication-defective FHV-1 vector-based vaccine platform that provides enhanced safety. Our results demonstrate that the replication-defective FHV-1, created by deleting the gL gene, exhibits favorable safety and induces protective immunity against FHV-1 challenges. More importantly, this virus shows an excellent ability to deliver VP2 of FPV, stimulating high levels of NAbs and protective immunity, highlighting its potential for further development.

The development of replication-defective herpesviruses through the deletion of replication-essential genes has been actively pursued. Notable examples include the deletion of immediate-early genes, such as ICP4 and ICP27 (29, 34), as well as the gD gene (26, 30). Since overexpression of gD and gH inhibits viral entry, a gD-deletion virus was rescued using a cell line (VD60) that expresses an inducible gD. In contrast, overexpression of gB and gL does not interfere with viral entry. During our study, we noted that other research reported that a replication-defective cytomegalovirus (a beta-herpesvirus) generated by gL deletion is a viable vaccine candidate (35). However, previous studies have shown that not all gL genes in herpesviruses are essential. For example, bovine herpesvirus 4, a gammaherpesvirus, retains its replicative capacity even after gL deletion. The essential role of gL in the replication of alphaherpesviruses has not been thoroughly investigated. In our study, we found that the one-step and multi-step growth curves of the wild-type virus showed no significant differences between the two cell lines, indicating that overexpression of gL does not affect viral titer. But we observed that the wild-type virus formed larger plaques in F81-gL cells compared to wild-type cells, suggesting that gL may promote the cell-entry process. Furthermore, rQD1 ΔgL-based viruses were only able to replicate in a single cycle in wild-type F81 cells, confirming that gL is an essential gene for FHV-1. These characteristics make gL an ideal target for constructing a replication-defective clone of FHV-1. Our data also demonstrated that rQD1 ΔgL-VP2 grows normally in the F81-gL cells.

Notably, the replication-defective rQD1 ΔgL-based viruses used in this study, administered via two intramuscular immunizations, induced exceptionally high levels of NAbs against FPV (up to 18 log2). Related studies have constructed attenuated live vector platforms, namely FHV ΔgI/gE/TK FCV VP1-FPV VP2 and WH2020-ΔTK/gI/gE-VP2, both of which incorporated deletion of the TK virulence gene and replacement with an FPV VP2 expression cassette (36, 37). FHV ΔgI/gE/TK FCV VP1-FPV VP2 (administered via a combination of intranasal and subcutaneous routes in a two-dose regimen) induced NAbs against FPV at approximately 6 log2. WH2020-ΔTK/gI/gE-VP2 (a single subcutaneous immunization at 10^8^ TCID_50_/mL) induced NAbs against FPV at approximately 8 log2. The differences in the levels of induced NAbs may be due to factors such as the gene insertion site and administration route. It is particularly noteworthy that all cats immunized with rQD1 ΔgL-based viruses developed anti-FPV NAbs within the first week post-immunization. Collectively, these findings demonstrate the superior capability of rQD1 ΔgL-based viruses in inducing anti-FPV VP2 NAbs. The underlying mechanism requires further investigation.

Herpesvirus glycoproteins gI and gE form heterodimers that participate in the secondary envelopment process of the virus, facilitating cell-to-cell spread and contributing to viral immune evasion (38–41). Although gI/gE is not essential for the replication of alphaherpesviruses, it plays a significant role in viral pathogenicity. Deletion or mutation of gI or gE genes in viruses such as VZV, HSV-1, pseudorabies virus, DEV, and FHV-1 results in varying degrees of attenuation in virulence (42–48). We found that the triple vaccine in which gI/gE was replaced by the VP1 cassette induced notably higher levels of NAbs against the VP2 protein of FPV, reaching titers as high as 18 log2. Moreover, the level of anti-VP2 NAb induced by 10^6.5^ TCID_50_ of rQD1 ΔgL/gI/gE-VP2-VP1 was even higher than that induced by 10^7.5^ TCID_50_ of rQD1 ΔgL-VP2. Thus, we speculate that gI/gE may contribute to the regulation of antibody response, although individual variations among cats cannot be ruled out. Further investigations will be needed to elucidate this phenomenon.

In this study, vaccination with 10^6.5^ TCID_50_ of the vaccine elicited NAbs against FCV VP1. However, the resulting titers were relatively low and did not meet expected levels. Previous studies have shown that live vaccines with the VP1 expression cassette inserted into the gI/gE locus can induce desirable NAb levels (37). The recombinant virus FHV ΔgI/gE/TK FCV VP1-FPV VP2 was administered in a prime-boost regimen over 42 days via combined intranasal and subcutaneous routes, inducing anti-FCV NAbs at approximately 12 log2. The primary immunization (intranasal) did not effectively induce anti-FCV NAbs by day 21, whereas the secondary immunization (combined intranasal and subcutaneous) significantly increased these antibody levels. These data indicate that to enhance the efficacy of rQD1 ΔgL-based viruses in delivering FCV VP1, further investigation into factors such as the insertion site and immunization route is necessary.

In summary, the replication-defective FHV-1 ΔgL platform offers a significant safety advantage and delivers foreign protective antigens against major feline pathogens, including FPV, FCV, and shows promise for targeting other pathogens such as Chlamydia felis, feline leukemia virus, and Leptospira felis. Combining this platform with the F81-gL-Rosa26 cell line, which is engineered for clinical safety, accelerates the translation of these vaccine candidates toward clinical evaluation and facilitates their route to market. Furthermore, this platform could potentially enhance vaccine immunoprotection by delivering molecular adjuvants, such as granulocyte colony-stimulating factor and the TLR9 agonist CpG49 (49, 50). Moreover, developing replication-defective vaccines for other herpesviruses could benefit from the data in the current study.

## MATERIALS AND METHODS

### Viruses, cells, and plasmids

The FHV-1 QD1 strain was isolated in 2020 from a clinical sample collected from a cat exhibiting ocular and nasal discharge and conjunctivitis in Shandong Province, China (Che, X. X., Huang, J. B., Li, L. P., & Lin, W. W. unpublished data). The FPV SR strain was isolated in 2022 from a clinical sample of a cat with diarrhea in Jiangxi Province, China (unpublished data). The FCV HBDL2 strain was isolated in 2021 from a clinical sample obtained from a cat showing severe systemic signs in Heilongjiang Province, China (51). Feline kidney cells (F81) and human embryonic kidney cells (293T) were maintained in our laboratory. Three F81-derived cell lines (F81-gL, F81-gL-Rosa26, and F81-gL-Random), stably expressing the FHV-1 gL gene, were established in this study. The recombinant plasmid pLVX-gL His-IRES-mCherry was synthesized by Nanjing Genscript Biotechnology. Other plasmids used in this study, including pUC19, pMD2.G, psPAX2, pcDNA3.1-eGFP-FPV VP2 (the VP2 gene codon-optimized for the feline system by Nanjing Genscript Biotechnology), pVAX1-VP1 (the VP1 gene codon-optimized for the feline system by Nanjing Genscript Biotechnology), lentiCRISPR v2, and pcDNA3.1-Cre, were maintained in our laboratory.

### Cell lines generation

Laboratory-stage line (F81-gL): This line was constructed by transfecting 293T cells with the lentiviral plasmid pLVX-gL His-IRES-mCherry and helper plasmids (pMD2.G and psPAX2). The harvested lentiviral particles were used to transduce F81 cells. Stable cells were established by fluorescence-activated cell sorting (FACS) of mCherry-positive cells and subsequent expansion.

Clinical production cell lines (safety-optimized): F81-gL-Rosa26 and F81-gL-Random were established in this study. Site-specific integration cell line (F81-gL-Rosa26): Using NCBI, the sequence of the murine commonly used safe-harbor locus, Rosa26, was analyzed via BLAST against the domestic cat genome to identify homologous feline Rosa26 sequences. Approximately 1,000 bp of flanking sequence on each side was obtained. This region was further confirmed by PCR amplification and sequencing using genomic DNA extracted from F81 cells. A gL-puromycin expression cassette was inserted into this locus. A targeting vector was constructed by cloning homology arms of 1,000 bp each upstream and downstream of the integration site, along with the expression cassette, into a pUC19 backbone, resulting in the recombinant plasmid pUC19-Rosa26-LR-gL His-IRES-Puro. Simultaneously, a lentiCRISPR v2-sgRNA-Feline Rosa26 plasmid was constructed, incorporating the sgRNA sequence (CGCTGCTTCTCGATTGTGGG) targeting the feline Rosa26 locus. These two recombinant plasmids were co-transfected into F81 cells. Stable transfectants were selected via stepwise puromycin treatment, followed by isolation of single-cell clones using FACS and expanded. Random integration cell line (F81-gL-Random): The gL-puromycin expression cassette was cloned into pUC19. The resulting plasmid (pUC19-gL His-IRES-Puro) was linearized and transfected into F81 cells. Stable cells were selected with puromycin, followed by FACS-based isolation of monoclonal lines and subsequent expansion.

### CRISPR/Cas9, homologous recombination (HR), and Cre-LoxP-mediated engineering of recombinant FHV-1

The FHV-1 gL gene was knocked out using CRISPR/Cas9 and HR. A recombinant plasmid, pUC19-gL-LR-eGFP-FPV VP2, was constructed via homologous recombination enzyme (Vazyme Biotech) using fragments A (2,641 bp), B (1,020 bp), C (1,020 bp), and D (3,642 bp), each containing 20 bp homologous arms between adjacent fragments. Fragment A is a linearized pUC19 plasmid. Fragments B and C are the left and right homologous arms (each 1,000 bp) of the gL gene, which were amplified by PCR from viral DNA. Fragment D is an eGFP-FPV VP2 expression cassette, flanked by LoxP sites on both sides of the eGFP sequence, which was amplified by PCR using the pcDNA3.1 eGFP-FPV VP2 plasmid as a template. pUC19-gL-LR-eGFP-FPV VP2 and lentiCRISPR v2-sgRNA (targeting the gL gene: AGCAGATTGTAACCCACCGG) were co-transfected into F81-gL cells (at 90% confluence) using PEI transfection reagent (Beyotime Biotechnology). At 6–12 h post-transfection, FHV-1 QD1 (10^8^ TCID_50_/mL) was inoculated into the cells at a virus-to-supernatant volume ratio of 1:1,000. After 48 h, the mixture of recombinant and wild-type viruses was harvested by three freeze-thaw cycles at −80°C followed by low-speed centrifugation. The mixture was serially diluted 10-fold and inoculated onto F81-gL cells. After 1 h of adsorption, the supernatant was removed and replaced with a mixture of 2× DMEM and 2% low-melting-point agarose (pre-warmed to 37°C). The cells were then inverted and incubated at 37°C. After 48 h, green fluorescent plaques were marked under an inverted fluorescence microscope and picked for inoculation into F81-gL cells. The recombinant fluorescent virus, rQD1 ΔgL-eGFP-VP2, was obtained after three rounds of plaque purification. To remove the eGFP marker, pcDNA3.1-Cre was transfected into F81-gL cells, followed by infection with rQD1 ΔgL-eGFP-VP2 at 24 h post-transfection. The mixture of recombinant fluorescent and non-fluorescent viruses was harvested at 72 h post-infection, and three rounds of plaque purification were performed as described above to obtain the non-fluorescent recombinant virus rQD1 ΔgL-VP2. Using the same strategy, the gI/gE genes of rQD1 ΔgL-VP2 were targeted for knockout and replaced with the eGFP-FCV VP1 expression cassette, resulting in the construction of the recombinant fluorescent virus rQD1 ΔgL/gI/gE-eGFP-VP2-VP1. The eGFP gene was subsequently excised using the Cre recombinase, ultimately yielding rQD1 ΔgL/gI/gE-VP2-VP1.

### Assessment of the stability of rQD1 ΔgL-VP2 and rQD1 ΔgL/gI/gE-VP2-VP1

The rQD1 ΔgL-VP2 and rQD1 ΔgL/gI/gE-VP2-VP1 viruses were serially passaged up to passage 20 (P20). PCR amplification was performed to identify the gL and VP2 genes in the rQD1 ΔgL-VP2 genome using two primer pairs (gL F/R: 5′-TTCGTTTTATTACTCTTGAAGATTTAT-3′ and 5′-AAGCTTTATACTATCTTGTAGAGA-3′; VP2 F/R: 5′-ATGTCCGACGGCGCCGTGCAGCCCGA-3′ and 5′-TCAGTACAGCTTTCTGGGGGCCAGC-3′). Similarly, two primer pairs were used to detect the VP1 and gI/gE genes in the rQD1 ΔgL/gI/gE-VP2-VP1 genome (VP1 F/R: 5′-ATGGCCGACGACGGCAGCATCACCAC-3′ and 5′-TCACAGCTTGGTCATCACGCTCCGGAT-3′; gI-gE F/R: 5′-ATGTCGTCGATAGCCTTCATCTATATA-3′ and 5′-AACTACGCGACTGTAATCTGGAGGA-3′). The PCR products of the VP2 and VP1 genes from the P20 generation of the recombinant virus rQD1 ΔgL/gI/gE-VP2-VP1 were sequenced. For Western blot analysis: the primary antibodies included a mouse anti-VP2 monoclonal antibody (dilution 1:2,000), a mouse anti-VP1 monoclonal antibody (dilution 1:2,000) raised in our lab, and a commercial mouse anti-GAPDH monoclonal antibody (dilution 1:2,000; Abbkine) as a loading control. The secondary antibody was DyLight 800-conjugated goat anti-mouse IgG (dilution 1:15,000; Abbkine). For indirect IFA, the primary antibodies included mouse anti-VP2 monoclonal antibody (dilution 1:500) or mouse anti-VP1 monoclonal antibody (dilution 1:500). The secondary antibody was DyLight 488-labeled goat anti-mouse IgG (dilution 1:1,000; Abbkine).

### Determination of viral titer and growth curve

Referring to the previously established method (51), the viral stock was subjected to 10-fold serial dilution and inoculated onto cells seeded at 90% confluency in 96-well plates. After 48 h, CPE were observed, and the viral titer (TCID_50_) was calculated using the Reed-Muench method. For the determination of one-step and multi-step growth curves, cells seeded at 90% confluency in 12-well plates were infected at MOIs of 0.01 and 1, respectively. For the multi-step growth curve, viruses were harvested at 6, 12, 24, 36, 48, 60, and 72 h post-infection, and the TCID_50_ at each time point was determined. For the one-step growth curve, viruses were harvested at 6, 12, 24, 36, 48, and 60 h post-infection, and the corresponding TCID_50_ values were measured. The growth curves were subsequently plotted.

### Examination by transmission electron microscopy

Virus-infected cells were cultured until the appearance of CPE. The cells were first fixed using a dual fixation process with glutaraldehyde and osmium tetroxide to preserve cellular ultrastructure. This was followed by dehydration through a graded ethanol series, infiltration with resin, and embedding and polymerization to form rigid blocks. The samples were then sectioned into ultrathin slices of tens of nanometers using an ultramicrotome. The sections were doubly stained with uranyl acetate and lead citrate. Finally, the sections were examined under a transmission electron microscope.

### Vaccination and virus challenge

In accordance with the methodology in our previous study (52), 21 healthy 2-month-old cats were randomly divided into three groups and vaccinated with different doses of rQD1 ΔgL-VP2 (10^7.5^ TCID_50_, *n* = 6; 10^6.5^ TCID_50_, *n* = 5; 10^5.5^ TCID_50_, *n* = 5) and one control group receiving DMEM (*n* = 5). A booster immunization was administered 21 days later via intramuscular injection with a volume of 1 mL. The total immunization period was 42 days. During this period, body temperature fluctuations were monitored every 3 days, ocular and nasal swabs were collected every 3 days for qPCR detection of viral shedding, and body weight changes were recorded weekly. At 15 days post-primary immunization, one cat from the 10^7.5^ TCID_50_ vaccination group was randomly selected and euthanized for PCR detection (using gD primers F: 5′-ATGATGACACGTCTACATTTTTGGTG-3′ and R: 5′-TTAAGGATGGTGAGTTGTATGTATT-3′) of tissues including heart, liver, spleen, lung, kidney, brain, nasal turbinate, throat, trachea, ischiatic nerve, and trigeminal nerve. Among these, the heart, liver, spleen, lung, kidney, brain, nasal turbinate, throat, and trachea were also subjected to histopathological analysis. Serum samples were collected via venipuncture on days 0, 21, and 42 for NAb detection. After the immunization period, an FHV-1 QD1 challenge was conducted. Each cat was inoculated with 0.5 mL of virus dilution (10^5.5^ TCID_50_/mL) via intranasal (0.2 mL per nostril) and ocular (0.05 mL per eye) routes. The challenge phase lasted 15 days, during which clinical signs were recorded and scored daily, body temperature was monitored daily, and body weight was measured every 3 days. Ocular and nasal swabs and anticoagulated blood were collected every 3 days for qPCR-based viral load quantification. On day 15 post-challenge, all cats were euthanized. The brain, nasal turbinate, throat, trachea, lung, sciatic nerve, and trigeminal nerve were harvested for nucleic acid extraction and subsequent qPCR analysis of viral load. Additionally, tissues including the brain, nasal turbinate, throat, trachea, and lung were also processed for histopathological analysis.

Based on the aforementioned procedures, 30 cats were randomly divided into two groups: one group received the rQD1 ΔgL/gI/gE-VP2-VP1 (10^6.5^ TCID_50_/mL, 1 mL/cat, *n* = 15), and the other group received DMEM as a control (1 mL/cat, *n* = 15). A booster immunization was given 21 days later, resulting in a total immunization period of 42 days. During this phase, body temperature fluctuations were monitored every 3 days, body weight changes were recorded weekly, and venous blood samples were collected every 7 days to isolate serum for detecting NAbs. After the immunization was completed, both the vaccinated groups and the DMEM control group were randomly divided into three subgroups: three immunized subgroups and three non-immunized subgroups. Each subgroup consisted of five cats and was designated for challenge experiments using the FHV-1 QD1, FPV SR, and FCV HBDL2 strains, respectively. The FCV HBDL2 strain (51, 53), FHV-1 QD1 strain (unpublished data), and FPV SR strain (unpublished data) were isolated and characterized in our laboratory. The challenge period for all viruses was 15 days. The FHV-1 challenge protocol was consistent with that described above. For the FPV SR challenge, the dose was 10^8^ TCID_50_/mL, administered orally at 1 mL per cat. For the FCV HBDL2 challenge, the dose was 10^8^ TCID_50_/mL, delivered intranasally (0.2 mL per nostril) and ocularly (0.05 mL per eye), totaling 0.5 mL. Clinical signs were recorded and scored daily for FHV-1 (Fig. 2A), FPV (Fig. 5A), and FCV. FCV clinical scores were determined based on previous studies (52). Body temperature fluctuations were monitored daily, and body weight changes were recorded every 3 days. Ocular and nasal swabs (for FHV-1 and FCV challenges) or anal swabs (for FPV challenges) were collected every 3 days. Anticoagulated blood was also collected every 3 days for qPCR detection of viral load (for FCV and FPV challenges). On day 15, all cats were euthanized, and tissue samples were collected for nucleic acid extraction and qPCR quantification of viral load (FHV-1 challenge: brain, nasal turbinate, throat, trachea, lung, sciatic nerve, trigeminal nerve; FPV challenge: heart, liver, spleen, lung, kidney, brain, duodenum, jejunum, ileum, colon, mesenteric lymph nodes; FCV challenge: nasal turbinate, throat, trachea, lung). Additionally, selected tissues (FHV-1 challenge: nasal turbinate, throat, trachea, lung; FPV challenge: duodenum, jejunum, ileum, colon, mesenteric lymph nodes; FCV challenge: nasal turbinate, throat, trachea, lung) were subjected to histopathological analysis. Euthanasia was performed by intravenous administration of 20% sodium pentobarbital (0.3 mL/kg), in accordance with the protocol recommended by the World Society for the Protection of Animals, as detailed in the “Methods for the Euthanasia of Dogs and Cats” guide. For qPCR detection (Vazyme Biotech) in these animal trials, the total DNA or RNA template input was 20 ng for each swab and anticoagulated blood sample, and 100 ng for each tissue sample. The primers and probes used in the qPCR assays were as follows: for FHV-1 (targeting the CIRC gene), the forward primer was 5′-ATGGGAGGAACTGCATCGAC-3′, the reverse primer was 5′-GTGATTCGTTGGCGTCCGTA-3′, and the probe was FAM-CCTACTGGATGGGATCGCCGGGCG-BHQ1. For FPV (targeting the NS1 gene), the forward primer was 5′-CCAGAAACCGTTGAAACCACAG-3′, the reverse primer was 5′-TGTGCCATCATTTCAATATAACTATCTGG-3′, and the probe was VIC-CAGCACAGGAAACAAAGCGCGGGAGAAT-BHQ1. For FCV (targeting the P76/VP1 gene), the forward primer was 5′-AATTTAATGGTGTGGAGGCGCG-3′, the reverse primer was 5′-TGGGGATCCCACCCATAATATTT-3′, and the probe was VIC-AGCATGTGCTCAACCTGCGCTAACGTGC-BHQ1.

### NAb assay

The serum samples were subjected to twofold serial dilution and then mixed with the virus (2,000 TCID_50_/mL) at a 1:1 ratio by vortex oscillation. After incubation at 37°C for 1 h, the mixture was inoculated onto F81 cells (90% confluency) in a 96-well plate. Following a 1 h adsorption period, the serum-virus mixture was removed and replaced with fresh maintenance medium (1% FBS). After 48 h, CPE were observed. While CPE in FHV-1- and FCV-infected cells could be readily identified by visual inspection, no distinct CPE was observed in FPV-infected cells. Therefore, IFA was employed to determine the presence or absence of CPE, using a monoclonal antibody against the VP2 protein (dilution 1:500) as the primary antibody and DyLight 488-labeled goat anti-mouse IgG (dilution 1:1,000; Abbkine) as the secondary antibody. Each serum dilution was tested in triplicate. A serum dilution was considered to exhibit complete virus-neutralizing capability only when no CPE was observed in all three replicate experiments.

### Statistical analysis

The data are presented as the mean ± standard deviation. Statistical significance was assessed via unpaired *t*-tests or one-way analysis of variance with Tukey’s multiple-comparison test conducted using Prism version 9.3.0 software (GraphPad Software), with a value of *P* < 0.05 considered indicative of a significant difference (*, *P* < 0.05; **, *P* < 0.01; ***, *P* < 0.001; ****, *P* < 0.0001), while *P* ≥ 0.05 was considered statistically non-significant (ns). “N/A” indicates that statistical analysis was not performed due to an insufficient sample size.

## Data Availability

All data needed to evaluate the conclusions in the paper are present in the paper, and additional data related to this paper are available upon request.
